# Household Air Pollution in Low- and Middle-Income Countries: Health Risks and Research Priorities

**DOI:** 10.1371/journal.pmed.1001455

**Published:** 2013-06-04

**Authors:** William J. Martin, Roger I. Glass, Houmam Araj, John Balbus, Francis S. Collins, Siân Curtis, Gregory B. Diette, William N. Elwood, Henry Falk, Patricia L. Hibberd, Susan E. J. Keown, Sumi Mehta, Erin Patrick, Julia Rosenbaum, Amir Sapkota, H. Eser Tolunay, Nigel G. Bruce

**Affiliations:** 1 *Eunice Kennedy Shriver* National Institute of Child Health and Human Development, National Institutes of Health, Bethesda, Maryland, United States of America; 2 John E. Fogarty International Center, National Institutes of Health, Bethesda, Maryland, United States of America; 3 National Eye Institute, National Institutes of Health, Bethesda, Maryland, United States of America; 4 National Institute of Environmental Health Sciences, National Institutes of Health, Research Triangle Park, North Carolina, United States of America; 5 National Institutes of Health, Bethesda, Maryland, United States of America; 6 MEASURE Evaluation Project at the Carolina Population Center and the Department of Maternal and Child Health, Gillings School of Global Public Health, University of North Carolina at Chapel Hill, Chapel Hill, North Carolina, United States of America; 7 School of Medicine, Johns Hopkins University, Baltimore, Maryland, United States of America; 8 Office of Behavioral and Social Sciences Research, National Institutes of Health, Bethesda, Maryland, United States of America; 9 Office of Noncommunicable Diseases, Injury and Environmental Health, Centers for Disease Control and Prevention, Atlanta, Georgia, United States of America; 10 Division of Global Health, Massachusetts General Hospital, Boston, Massachusetts, United States of America; 11 Palladian Partners, Silver Spring, Maryland, United States of America; 12 Global Alliance for Clean Cookstoves, Washington, District of Columbia, United States of America; 13 Women's Refugee Commission, New York, New York, United States of America; 14 FHI 360, Washington, District of Columbia, United States of America; 15 School of Public Health, University of Maryland, College Park, Maryland, United States of America; 16 Division of Cardiovascular Sciences, National Heart, Lung, and Blood Institute, National Institutes of Health, Bethesda, Maryland, United States of America; 17 Department of Public Health and Policy at the University of Liverpool, Liverpool, United Kingdom and the Department of Public Health and Environment at the World Health Organization, Geneva, Switzerland

## Abstract

William Martin and colleagues report on their stakeholder meetings that reviewed the health risks of household air pollution and cookstoves, and identified research priorities in seven key areas.

*Please see later in the article for the Editors' Summary*

**Summary Points**

- Household air pollution (HAP) from solid fuel (biomass or coal) combustion is the leading environmental cause of death and disability in the world.
- Many governments, multinational companies and nongovernmental organizations are developing programs to promote access to improved stoves and clean fuels, but there is little demonstrated evidence of health benefits from most of these programs or technologies.
- A stakeholder meeting hosted by U.S. government sponsors identified research gaps and priorities related to the health effects of HAP and unsafe stoves in seven areas (cancer; infections; cardiovascular disease; maternal, neonatal, and child health; respiratory disease; burns; and ocular disorders) and gaps in four cross-cutting areas that are relevant to research on HAP (exposure and biomarker assessment, women's empowerment, behavioral approaches, and program evaluation).
- It is vital that researchers partner with implementing organizations and governments to evaluate the impacts of improved stove and fuel programs to identify and share evidence regarding the outcomes of the many implementation programs underway, including the socio-behavioral aspects of household energy use.

## Introduction

Household air pollution (HAP), which results from incomplete combustion of the solid fuels traditionally used for cooking and heating, affects the homes of nearly 3 billion people. It is the leading environmental cause of death and disability worldwide, with highest risks for women and children due to their domestic roles [1]. The high levels of pollutants found in HAP cause a range of diseases [1], in addition to burns and scalds [2] and injuries or violence experienced during fuel collection [3]. Additionally, household solid fuel use can pose substantive environmental risks, including degradation from fuel gathering as well as climate change from release of both CO_2_ and short-lived climate forcers, such as black carbon, during combustion [4]. Despite the broad support to find solutions, only a few solid fuel interventions have shown that they might improve health over the long term [5]–[7], especially when implemented at the scale required (Box 1).

**Box 1. Finding Household Energy Solutions—The Context**

To be successful, efforts to introduce improved stoves and fuels must take into account the scale of the problem as well as the complex social, environmental, and economic context of HAP:

- **Scale of the problem:** Nearly 3 billion people use unsafe and inefficient traditional stoves and fuels for cooking and heating.
- **Gender- and age-specific risks:** Women and children have the greatest exposures to HAP and unsafe stoves but may be constrained by cultural and gender-related factors to change their household exposures and risks with fuel-gathering.
- **Cultural contexts:** Traditional methods of cooking and heating have been used for many generations and are adapted to local dietary, environmental, and cultural needs.
- **Environmental risks:** Household fuel combustion contributes to outdoor air pollution and climate change and, in some regions, fuel-gathering for inefficient stoves contributes to environmental degradation, including deforestation and desertification.
- **Poverty:** Solid fuel use is closely linked to poverty both within and between countries, and clean cooking technologies must be affordable and desirable to families with limited and often insecure incomes to provide sustainable solutions.

Using data from 2004, the World Health Organization (WHO) estimated that nearly 2 million premature HAP-related deaths occurred from acute lower respiratory infections (ALRI) in young children, chronic obstructive pulmonary disease (COPD), and lung cancer [8]. The recent Global Burden of Disease 2010 update by the Institute for Health Metrics and Evaluation nearly doubles the estimated mortality to 3.5 million (4 million including HAP's contribution to 16% of outdoor air pollution deaths), due to the inclusion of HAP deaths from cardiovascular disease and lung cancer from biomass smoke [1]. Prenatal exposure to HAP is linked to the increased risk of stillbirth, low birth weight [9], and impaired cognitive development [10], and direct HAP exposure is linked to cataracts [11] and possibly trachoma [12],[13].

In line with the United Nations (UN)-led initiative, Sustainable Energy for All (SEFA)—an ambitious campaign to bring modern energy to every home by 2030 [14]— governments, multinational companies and nongovernmental organizations (NGO) are increasing investments in programs to promote access to improved stoves and clean fuels that could mitigate these harmful effects. Much of this effort is facilitated by the United Nations Foundation's Global Alliance for Clean Cookstoves (Alliance) [15], which has the goal that 100 million homes adopt clean stoves and fuels by 2020. Furthermore, agreement on a set of voluntary, tiered standards for stove performance [16] and new WHO indoor air pollution guidelines for household fuel combustion expected to be published in 2013 [17] will allow consumers and those implementing these efforts to know, for the first time, the emissions and potential health impacts of a given stove. Because momentum to improve stoves and household air quality is growing rapidly, evaluation of the impact of household energy interventions on health is both urgent and essential [18].

Programs to introduce clean cookstoves cannot simply assume that these so-called improved stoves will be accepted by the household or that they will benefit health. Forgoing a thorough evaluation during the initial stages of implementation and scale-up, particularly of the stove's acceptability and performance in everyday use, carries the risk that implementation will not improve health [19]. In addition, preliminary exposure–response results from the recent RESPIRE trial suggest that stoves must significantly reduce exposures (by at least 50%) to substantially improve health [20]. If this finding is replicated in future studies, the daunting task ahead is to facilitate access to cleaner-burning stoves and fuels that are affordable, acceptable to families, and scalable to hundreds of millions of households.

This report results from an international meeting of experts in research, technology, and development, hosted by the National Institutes of Health (NIH) and other U.S. government partners, that led to a proposed research agenda to address the gaps in the current evidence on the health effects of HAP and unsafe stoves and the identification of critical considerations for effective implementation (Box 2). The imminent scale-up of stove and fuel improvement programs offers a great opportunity for health researchers to work with program implementers. Failure to do so risks introducing new cooking technologies to millions of homes without understanding whether the intended health benefits are realized—or worse, whether there are unintended adverse consequences.

**Box 2. Process for Developing HAP Health Research Recommendations**

**Participants**

The recommendations presented in this report result from a workshop involving 139 participants (See Supplement S1 for agenda and participant list):

- Participants were from 15 nations
- Participants included 8 members of the expert group on HAP for the new Global Burden of Disease project comparative risk assessment 2010 update [1],[48]

**Topics**

Working groups examined:

- A set of health outcomes
  - Cancer
  - Infections
  - Cardiovascular disease
  - Maternal, neonatal, and child health
  - Respiratory disease
  - Burns
  - Ocular disorders
- A set of cross-cutting considerations for health research on improved stoves and fuels
  - Exposure and biomarker assessment
  - Women's empowerment
  - Behavioral approaches
  - Program evaluation
- Health research strategies needed to fill knowledge gaps

**Process**

For each of the above topics, working groups identified health research gaps and crafted recommendations through group consensus via the following process:

- Pre-workshop: Drafted 10 white papers. Drafts drew on:
  - Published systematic reviews (where available)
  - Additional recent primary publications identified through PubMed and ISI Web of Knowledge, references from identified papers, and working group members' knowledge of other published and unpublished studies
- During workshop: Further developed white papers.
  - Ten topic-specific working groups further developed the white papers
  - Working groups presented white papers in plenary sessions for further input
- Post-workshop: Drafted current paper as a result of a writing workshop with authors in October 2011. The organizing committee and working groups selected the authors by consensus to provide this summary report.

## Findings

We identified gaps in research relating to the health effects of unsafe stoves and fuels in seven disease areas (Table 1) and several cross-cutting considerations for all research on improved stoves and fuels (Table 2).

**Table 1:** Summary of major research gaps and needs for evidence on health outcomes.

Health Topic	Major Gaps and Needs Identified
Cancer	• Determine the risk from coal-related HAP exposure on cancer of organ systems other than the lung.• Assess the risk from biomass-related HAP exposure for cancer of the lung, upper airway, and other organ systems.• Investigate whether risk is mediated via germline, somatic, or epigenetic changes and whether there is a developmental window of susceptibility.
Infections	• Carry out population-based studies to determine the impact on important infectious diseases, including TB and malaria (the latter via effects of smoke on biting and disease transmission), and the impacts of interventions.• Extend the experience of the RESPIRE study on acute child pneumonia to other populations and cultures and determine etiology (pathogens) and exposure–response relationships more precisely.• Leverage existing epidemiologic studies investigating pneumonia and the impacts of new vaccines by adding HAP exposure assessment.
Cardiovascular disease	• Use short- and longer-term observational studies (including those leveraging existing cohorts) and intervention studies to determine the risk of completed cardiovascular outcomes, indicators of disease process (e.g., ECG findings), and risk (e.g., blood pressure, lipid levels, inflammatory biomarkers).• Determine the role of HAP in the developmental origins of CVD through long-term cohort studies.
Maternal, neonatal, and child health	• Strengthen existing evidence on pregnancy outcomes (pre-term birth, IUGR, stillbirth), with assessment of gestational age and vulnerable periods of exposure during pregnancy.• Investigate the risk of severe infection in neonates and young infants.• Strengthen emerging evidence on child growth and cognitive development to 5–7 years of age.• Determine the risk of HAP exposure for the main causes of maternal mortality and morbidity.• Establish long-term cohorts to study the role of early HAP exposure and associated mechanisms (including epigenetic) in the developmental origins of later childhood and adult disease.
Respiratory disease	• Use cohort studies and clinical trials to determine the roles of HAP in both causation and exacerbation of asthma in children.• Assess the impacts of HAP exposure reduction on the rate of lung function decline over the medium term (e.g., 5 years) in young/middle-aged women.• Describe the risks of HAP exposure in pregnancy and early life for lung development, asthma, and COPD.
Burns	• Enhance surveillance and population-based evidence on the causes, incidence and mortality, disability, and longer-term social impacts of burn injuries.• Assess the impact of safety testing of new stoves.• Determine the value of prevention strategies on morbidity and mortality related to burn injuries or accidental poisoning (e.g., with kerosene) from cooking, heating, and lighting.
Ocular disorders	• Extend the evidence on cataracts in men and in exposed populations outside of India.• Ensure better control of potentially serious confounding in studies of cataract (e.g., smoking, UV light exposure, nutrition).• Strengthen tentative evidence on risk for other important ocular disorders, such as trachoma.• Investigate the motivational potential of reduced eye symptoms (tearing, irritation) for intervention programs.

CVD, cardiovascular disease; ECG, electrocardiogram; IUGR, intrauterine growth restriction; TB, tuberculosis; UV, ultraviolet.

**Table 2:** Summary of major gaps and research needs for cross-cutting issues.

Health Topic	Major Gaps and Needs Identified
Exposure and biomarkers	• Better characterize spatial and temporal variability in exposures to HAP by studying critical behavioral patterns and individual- and household-level characteristics.• Further develop and field test small/light and highly time-resolved personal monitors for particulate matter and other important pollutants (e.g., size-specific and chemical constituents of particulate matter, carbon monoxide).• Develop standardized and comprehensive exposure-assessment protocols (including questionnaires to understand critical factors in exposure variability), suitable for use with intervention-evaluation and epidemiologic studies.• Develop and validate methods to estimate dose, including biomarkers of exposure, especially for cumulative exposures.• Assess the role of validated biomarkers of early effect or early disease activity in studies of chronic disease.
Women's empowerment	• In research and evaluation, include sex-disaggregated analysis and pay attention to gender dimensions of behaviors that affect the uptake of clean cooking interventions and the health risks associated with fuel collection.• In epidemiologic studies on health outcomes, recognize that women may not access health services with the same frequency as men, resulting in bias in studies from clinics and hospitals.• Assess the potential educational and economic benefits of improved stoves or fuels that provide more free time and reduced health risks for women and girls.
Behavioral change	• Ensure that behavioral research plays a more central role in stove and program design to optimize the safe and exclusive use of new stoves and clean fuels to minimize exposure and burn risks.• Evaluate behavior-change interventions for proper and exclusive use of improved stoves and fuels, exposure reductions, and safety improvements.
Program evaluation	• Strengthen cooperation between investigators and implementers to develop more appropriate study designs using standardized methods for assessing health impacts.• Make the results of evaluation available as rapidly as possible and in a manner that encourages widespread learning and quality improvement.

### Key Gaps in Health Research

Among the research gaps presented in Table 1, those relating to the highest burden (e.g., cardiovascular disease, child pneumonia) and outcomes linked to child survival and development are likely to have the most impact on generating awareness of the problem and in mobilizing international mitigation efforts and funding. The impact of HAP exposure during pregnancy and early infancy on the development of disease in later life is another emerging, high-priority topic, as is the prevention of burns, scalds, and poisoning, which has received far too little attention in the past. Nevertheless, securing strong and consistent evidence of the impact of HAP on other conditions, such as tuberculosis and eye diseases, e.g., cataracts, would not only extend the overall evidence and attributable disease burden linked to HAP exposure, but also have important implications for strategic priorities in the respective control programs. (The priorities outlined in Table 1 are further developed and available for review as part of the Roadmap Recommendations of the Alliance's Health Working Group [21].)

## Cross-cutting Considerations for Research

### Exposure and Biomarker Assessment

**Figure 1:**
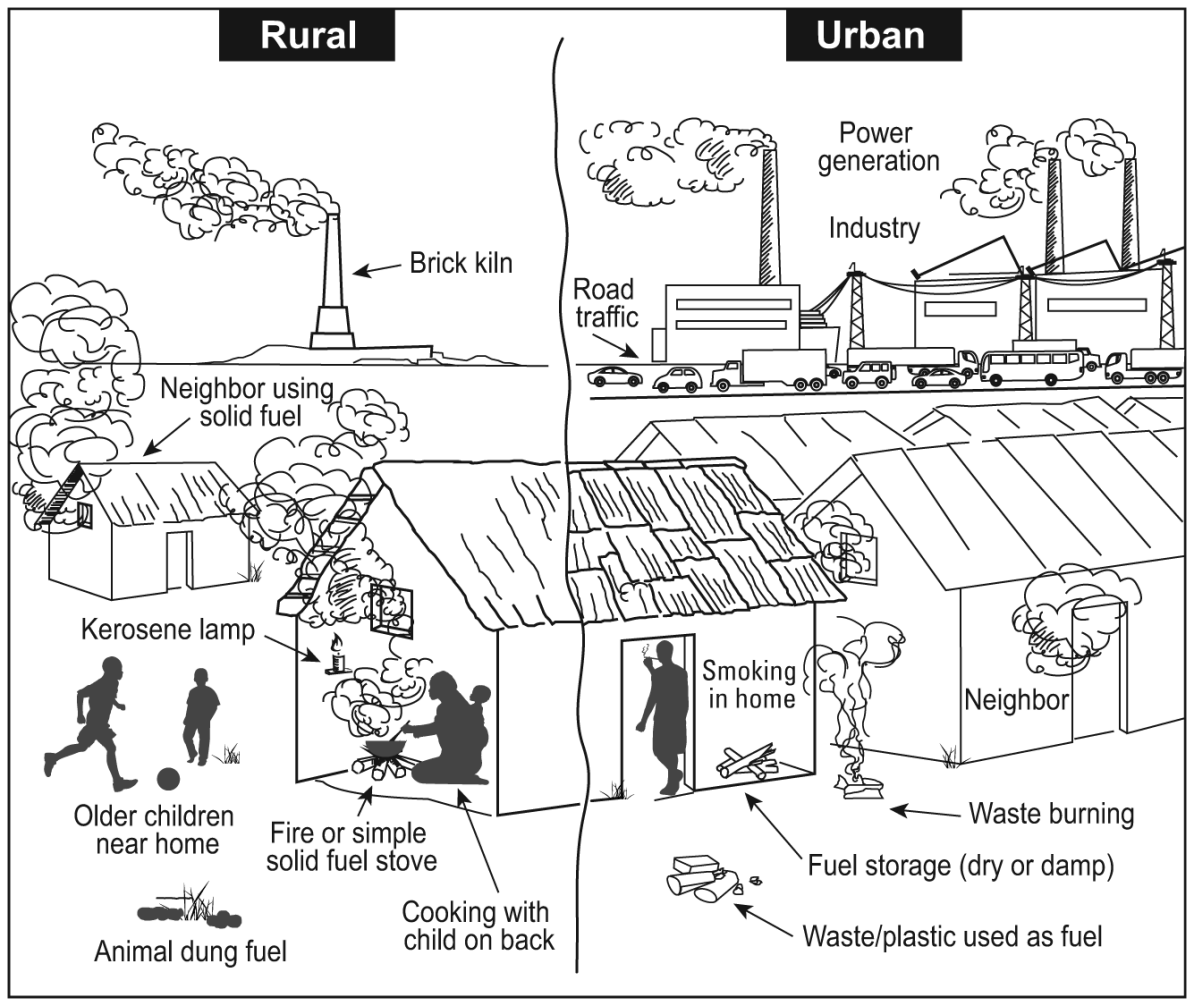
HAP in urban and rural settings with examples of other confounding sources of pollutants. Multiple factors influencing household air pollution and personal exposure levels need to be considered for effective measurement of exposure in health research and evaluation studies, which will differ in urban vs. rural settings and may vary based on cultural practices, geography, and elevation. Each site of HAP must be carefully assessed for other potential sources of products of incomplete combustion that may confound household or personal monitoring of exposure.

Exposure assessment must account for a complex set of factors (Figure 1) that result in large variations in actual exposure and dose through time, between individuals, and among settings. Exposure assessments used in cookstove studies have tended to use simple, often categorical or qualitative measures [22]–[25]. Because these measures cannot account for the high degree of uncertainty and variability in HAP exposures [26],[27], studies using these measures are limited in their ability to elucidate dose–response curves and to detect changes in health outcomes associated with differences in exposure with sufficient statistical power. Greater investment is needed to enhance the sophistication of exposure assessments, including more frequent and numerous samples and more rigorous characterization of the factors that influence exposure variability. Because the factors driving spatial and temporal variability are not identical across different locations, studies need to include the collection of relevant data for exposure variability and uncertainty for their study context, using consistent and compatible protocols, survey tools, and instrumentation.

Biomarkers (e.g., carboxyhemoglobin, polycyclic aromatic hydrocarbon urine metabolites, isoprostane [28], and lung macrophage carbon loading [29],[30]) have been employed to assess human doses, especially for short-term exposures. Affordable biomarkers that are validated to accurately reflect medium- to longer term exposure and are scalable for applications in both health research and program evaluation are a priority research need. Research may seek to extend knowledge on the potential application and usefulness of currently recognized biomarkers or develop and test new biomarkers. Biomarkers of effect and early stages of disease can also play a role in cookstove health studies, especially for chronic, latent health effects, for which these biomarkers may prove especially useful.

### Women's Empowerment

Women and girls in developing countries are usually responsible for cooking and fuel-gathering and thus experience the greatest exposures and HAP-related elevations of risk. Due to poverty and the perception of low opportunity costs for time spent fuel-gathering and cooking with inefficient stoves, women may be constrained in their ability to change this situation [31],[32]. In addition to the health risks from HAP exposure and burns, women and girls face hazards during fuel-gathering, including violence and injuries [3], and spend long hours at this task that may reduce engagement in educational or economic activities [33]. Future research must assess both the gender-specific risks of traditional stoves and fuels and the putative benefits of their improvement.

### Behavioral Approaches

Human behavior is critical to adopting clean, safe stoves and fuels, using them properly, and improving health outcomes. Behavioral research can determine the best ways to influence the attitudes and beliefs relevant to adopting and maintaining new stoves and fuels, identify the positive features of improved stoves—such as fuel savings—that promote adoption and sustained use, assist in the design of interventions, help ensure proper use of new technology, and improve the home environment [34]. Although research in this area is limited, behavioral changes have been proposed to reduce risks to children [35]. Additionally, evidence-based strategies to change behavior relating to water and sanitation [36] may be adaptable to reduce HAP exposure [33].

### Program Evaluation

Household energy interventions are “complex”, involving new technologies and fuels, promotion of behavioral changes, and institutional factors, including product supply and financing, all of which might be implemented through combinations of markets, governments, and NGOs. Not surprisingly, evaluation of intervention programs is far from straightforward. Various evaluation methods should be used to inform program design; examine whether stoves are adopted, used, and maintained; determine whether anticipated exposure reduction and consequent health benefits are realized; examine costs relative to benefits; and determine the reasons for a program's results.

Program evaluation that includes the measurement of impacts on health outcomes is needed to demonstrate the effectiveness of interventions at scale, but it will be resource-intensive. Consequently, such evaluation must be carefully planned to provide evidence on a relatively small but representative number of intervention types and settings. As measurement of exposure and biomarkers improves and exposure–response evidence is strengthened, these tools should provide a simpler, cheaper means of estimating health impacts on a large scale, as a complement but not a substitute for the direct measurement of health outcomes in some studies.

Because randomized controlled trials are difficult to implement in programmatic situations (especially where market-driven), program-appropriate evaluation designs capable of providing robust evidence are needed. Evaluators must overcome practical challenges, such as building political support for evaluation, balancing competing pressures, and managing the expectations of multiple stakeholders [37].

## Research Strategies

Three interrelated approaches are needed to address these research and evaluation priorities. The first approach is to focus research over 5 to 10 years on (i) establishing and quantifying risk where this is unknown or still uncertain and elucidating the mechanisms by which HAP results in disease; and (ii) strengthening and extending the description of exposure–response functions for some of the high-burden outcomes. Intervention-based research over this timeframe will be restricted to diseases with relatively short time intervals between exposure and effect, exacerbation of chronic disease, or markers of longer term disease development. Retrospective observational designs (e.g., case-control studies) can be used to investigate risk for established chronic disease over a short time frame, but not as a result of an intervention (examples in Table 3).

**Table 3:** Approaches and key study designs required to address research and evaluation priorities.

Nature of Research and Evaluation	Study Designs/Data Collection Methods	Examples of Research Areas for Which Approach Would Be Appropriate
Investigator initiated	Cohort studies (short term)	Risks of exposure for pregnancy outcomes, birth weight, and diseases in the neonate and young child; mechanisms
	Cohort studies (longer term)	Child growth and development, with follow-up into adulthood; chronic disease; developmental origins of adult disease
	Case control	Etiological studies, especially of rarer events (e.g., severe outcomes and mortality, congenital abnormalities) and chronic, longer-latency outcomes (e.g., cancer, IHD, eye disease, TB, CVD)
	Intervention: randomized including cluster and step-wedge designs	Impacts of interventions on mainly short-term outcomes and longer-term effects of differential exposure in pregnancy and early life, including pregnancy outcomes, child pneumonia, burns, and risk factors for chronic disease
Evaluation of implementation programs	Intervention: quasi-experimental	Earlier stages of implementation, e.g., impacts on HAP, exposure, burns
	Matched comparisons (randomization unlikely to be compatible with program implementation)	Shorter- to medium-term health outcomes
	Case control	Shorter- to medium-term health outcomes as programs reach scale
Routine data collection and surveillance	Surveillance	Shorter- to medium-term health outcomes as programs reach scale
	Registries	Etiological studies and evaluation of larger-scale interventions

CVD, cardiovascular disease; IHD, ischemic heart disease; TB, tuberculosis;

The second approach is to monitor longitudinal cohorts over longer periods and to assess the risks of HAP exposure on the development of chronic diseases. A range of study designs will be required over varying time scales (Table 3), and existing investments such as birth cohort studies [38] and large-scale intervention programs can be leveraged to support these long-term analyses.

The third strategy involves evaluating health impacts from large-scale introductions of improved stoves or fuels in real-world settings, using either randomized or non-randomized designs (Table 3). Because the rapid evolution of clean cookstove technology may result in replacement of an outdated stove in the middle of a long-term study, the success of such research will depend on stable, pre-defined standards for measuring pollution in the household environment and biomarkers in household members. This third approach is the most challenging as it requires commitment of partners that may have very different agendas. Evaluation will be greatly facilitated by cooperation with the programs concerned, but must be independent and avoid any conflicts of interest.

## Discussion

Now is a unique opportunity in time to determine key factors that can sustainably reduce exposure to HAP and improve health in low- and middle-income countries. To achieve synergy with the converging commitments of governments, funders, NGOs, and stove manufacturers to implement clean cooking solutions, research and evaluation must focus on priority areas (Tables 1 and 2), which include: (1) strengthening evidence across a range of health outcomes; (2) scalable applications of exposure monitoring and use of biomarkers; and (3) determinants of successful implementation programs, including socio-behavioral aspects of household energy use. Also included must be an awareness of and additional focus on those with the highest exposures: women and young children. The goal is the coordinated and timely use of research and evaluation to inform and, when needed, modify implementation programs to provide the best chance to help the most people in the shortest time possible.

### Lessons from the Past

The field of public health is littered with examples of failed interventions designed to improve human health [39]. Primary among these are those interventions that require substantial changes in human behavior to be successful [36]. Unless households adopt and use cleaner stoves and fuels that are capable of delivering sufficient exposure reductions, their health benefits will not be realized. Many factors influence adoption of clean energy solutions, which, at the household and community levels, include whether: (1) they are affordable and desirable to families, (2) women have decision-making influence, and (3) there is community involvement and support at the beginning of the intervention [33].

Despite the best intentions, interventions to improve health may not only be unsuccessful, but may have unintended, catastrophic consequences. An example is the wells installed in south Asia to provide access to clean groundwater and prevent the spread of cholera; they did not prevent cholera but did lead to widespread arsenic poisoning [40]. Improving indoor air quality is not likely to have such striking adverse impacts, as compliance with air quality standards has provided tremendous public health benefits [41]. It is commonly believed in malaria-endemic areas that indoor smoke wards off mosquitoes and could therefore reduce malaria transmission. Biran et al. notes, however, that while smoke may reduce bites, there is no evidence that cleaner indoor air promotes malaria [42]. Nonetheless, the message is clear: Independent evaluation of implementation programs not only provides the opportunity to confirm whether the primary program goals are realized; it also permits assessment of unexpected co-benefits or adverse outcomes.

The challenge is to develop a forum for implementers and investigators to share their perspectives and goals in a way that permits independent evaluation of the programs' health impacts and allows evaluators to propose modifications to improve outcomes.

### Design Challenges for New Research

Three unique challenges will be inherent to HAP studies. First, the proposed improved stove or fuel intervention must achieve a large and sustainable reduction in HAP [20]. Second, each household must be willing to (more or less) exclusively use the new improved stove or fuel, as shared use of the new and traditional stoves, or “fuel stacking”, is common [31] and is unlikely to result in sufficient reduction in exposure [43]. Studies must objectively assess stove use in practice, by using electronic devices such as “stove use monitors” (SUMs) [44], and conducting qualitative research to understand preferences and choices. Third, study budgets must adequately support thorough exposure measurement to ensure sustained reductions occur and to quantify exposure–response relationships.

### Building Capacity for New Investigators

Training and sharing of experience will help to effectively overcome these challenges. Furthermore, considering the range of research priorities and the pressing requirement for thorough program evaluation, new, multidisciplinary investigator teams will be essential. A small number of training sites already exist in host countries [45], while others are managed by NGOs [46]. In 2012, the Alliance announced its first RFA for health research related to HAP and child survival [47], which emphasizes strengthening of local capacity. Also in 2012, the NIH funded 14 competitive awards to supplement NIH grantees and hosted its first HAP training workshop for investigators. Despite these encouraging developments, substantial new investment will be necessary to build the capacity to carry out this research and evaluation agenda.

## Conclusions

Although nearly 3 billion of the world's poorest people still rely on household fuels and stoves that have changed little from prehistoric times, the international community is at last showing signs of a meaningful response. Major implementation programs are already underway to meet the Alliance's 100 million 2020 goal and the UN SEFA target of universal access by 2030. However, research and evaluation must be part of this global effort. It cannot simply be assumed that current efforts to encourage adoption of cleaner and more fuel-efficient stoves and fuels will deliver large health benefits. To secure these gains, programs require evidence-based technology and delivery mechanisms and robust, well-resourced, transparent, and timely evaluation. This report identifies research priorities for global efforts to implement effective clean cooking solutions, with important implications for disease control programs, exposure measurement and biomarker validation, behavioral considerations for effective adoption, and program evaluation. Well-planned investment, complemented by cooperation between the research and implementation communities on research, evaluation, and training, can fill these gaps and make an important contribution to improving health. The recent developments in energy access, described in the Introduction, provide the field with its first opportunity to mobilize and coordinate existing efforts by integrating research and training with practical solutions across various sectors to improve health and quality of life for millions, especially women and children living in poverty. Quickly demonstrating the beneficial health impacts of clean stoves in multiple settings could ensure the successful scale-up and funding of this critical health program.

## Supporting Information

Supplement S1 Agenda and participant list for the May 2011 workshop, “Health Burden of Indoor Air Pollution on Women and Children in Developing Countries.”
(DOC)

## Abbreviations

Alliance: Global Alliance for Clean Cookstoves
HAP: household air pollution
NGO: nongovernmental organization
SEFA: Sustainable Energy for All
UN: United Nations
WHO: World Health Organization
