# Supplementary material for: Household Air Pollution in Low- and Middle-Income Countries: Health Risks and Research Priorities
Source: PLoS Med. 2013 Jun 4;10(6):e1001455. doi: 10.1371/journal.pmed.1001455 (PMC3672215; doi:10.1371/journal.pmed.1001455)
Supplement: Supplement S1 — Agenda and participant list for the May 2011 workshop, “Health Burden of Indoor Air Pollution on Women and Children in Developing Countries.” (DOC) [file pmed.1001455.s001.doc]

# Health Burden of Indoor Air Pollution on Women and Children in Developing Countries (Final Agenda)

# May 9-11, 2011

| **Monday, May 9, 2011** | |
| --- | --- |
| 5:00 pm to 7:30 pm | **Registration** |
| 5:00-7:30 pm | **Evening Reception** (Crystal Room) |
| 7:30 pm – 8:00 pm | **Welcome and opening remarks (**Decatur/Farragut Rooms)  Francis Collins, Director, U.S. National Institutes of Health  Hillary Rodham Clinton, Secretary of State, U.S. Department of State (welcome video)  Kris Balderston, Special Representative for Global Partnerships, Office of the Secretary of State  Gina McCarthy, Assistant Administrator for Office of Air and Radiation, U.S. EPA |
| 8:00 pm – 8:40 pm | Pilar Nores de Garcia, Presidenta, Instituto Trabajo y Familia  “The Peruvian National Stove Program: the early successes for women and children in Peru” (20 minutes Q&A) |
| Tuesday, May 10, 2011 | |
| 7:00 am – 8:00 am | **Registration** |
| 8:00 am – 8:20 am | Welcome and Instructions to Workshop **(**Decatur/Farragut Rooms)  William J Martin II, Co-chair, Eunice Kennedy Shriver National Institute of Child Health and Human Development  John Balbus, Co-chair, National Institute of Environmental Health Sciences  Linda Birnbaum, Director, National Institute of Environmental Health Sciences  Alan Guttmacher, Director, Eunice Kennedy Shriver National Institute of Child Health and Human Development |
| 8:20 am – 8:40 am | **A decade of progress leading to the Global Alliance**  Jacob Moss, U.S. Department of State  Leslie Cordes, Global Alliance for Clean Cookstoves, United Nations Foundation |
| 8:45 am – 9:30 am | **Household air pollution in lower and middle income countries: the scope of the problem**  Kirk Smith, University of California, Berkeley |
| 9:30 am – 10:30 am | **Key issues Panel # 1** Chairs: William J Martin II and John Balbus  Subgroup representatives from Cancer, Respiratory, Cardiovascular, Burns and Ocular health; Q&A |
| 10:30 am – 10:45 am | Break |
| 10:45 am – 11:45 am | **Key issues Panel # 2** Chairs: William J Martin II and John Balbus  Subgroup representatives from Pregnancy/Newborn, Infections, Women’s Empowerment and Exposure/Biomarkers; Q&A |
| 11:45 am – 12 :00 pm | Break |
| 12:00 pm – 1:30 pm | **Women’s empowerment panel and discussion** (lunch). Anita Botti, Office of Global Women’s Issues, U.S. Department of State and Jamie Bechtel, New Course  Women’s role in family decision –making regarding stove purchase and use  Magi Matinga, University of Johannesburg, South Africa  Violence to women and children during fuel gathering  Erin Patrick, Women’s Refugee Commission  Economic opportunities for women in owning and managing local stove/fuel businesses  Veena Sharma, Self Employed Women's Association |
| 1:45 pm – 3:45 pm | **Break-out sessions for each working group to review and start revision of draft summaries of following topic areas (may include invited presentations):**  o Respiratory (James Conference Room)  o Infections (Dewey II Conference Room)  o Cancer (Charleston I Conference Room)  o Cardiovascular (Charleston II Conference Room)  o Pregnancy/Neonatology (Rappahannock Conference Room)  o Burns and Ocular health (Roanoke Conference Room)  o Women’s Empowerment (Dewey III Conference Room)  o Exposure Assessment and Biomarker Development (Dewey I Conference Room) |
| 4:00 pm – 5:30 pm | **Health assessment of major implementation programs panel and discussion (**Decatur/Farragut Rooms)  Chairs: Neal Brandes, United States Agency for International Development and Simon Bishop, Shell Foundation and Christa Roth, Food and fuel  Epidemiologic assessment and causal inference models in India  Kalpana Balakrishnan, Sri Ramachandra University  Use of matched pairs in conditional cash transfer in Mexico  Clayton Nall, Harvard University  Reflections on innovative evaluation tools  Sian Curtis, University of North Carolina |
| 6:00 pm – 8:00 pm | **Evening Reception** (Crystal Room and includes stove and air monitoring exhibits, photographic display from COPD Foundation, breakout session interim summaries) |
| Wednesday, May 11, 2011 | |
| 8:00 am – 10:00 am | **Continuation of break-out sessions improving draft white paper and identifying final areas of research gaps and opportunities** (same rooms as May 10) |
| 10:00 am – 10:15 am | Break |
| 10:15 am – 11:45 am | **Existing centers of excellence, programs and infrastructure: opportunity to leverage IAP health research panel and discussion (**Decatur/Farragut Rooms) Chairs: Alan Guttmacher, NICHD and Henry Falk, CDC   - NHLBI Collaborating Centers of Excellence Arun Chockalingam, NHLBI - NICHD Global Network Linda Wright, NICHD - Medical Education Partnership Initiative (MEPI) Yvonne Njage, Fogarty international Center - Bangladesh International Center for Diarrheal Disease Research Alam Dewan, ICDDRB - Partnership for Clean Indoor Air (PCIA) John Mitchell, EPA - CDC platform countries Robert Spengler, CDC |
| 11:45 am - 12:15 pm | Break |
| 12:15 pm – 2:15 pm | **Working Lunch / Final report back from each working group** Chairs: Yvonne Njage, Fogarty International Center and Nigel Bruce, World Health Organization and University of Liverpool |
| 2:15 pm – 2:30 pm | Break |
| 2:30 pm – 3:30 pm | **Discussion of critical research gaps or opportunities in response to the “report back” by working groups**  Chairs: William J Martin II and John Balbus |
| 3:30 pm – 4:00 pm | **Expert panel review and summary**  Joseph Graziano, Columbia University  Judith Palfrey, Harvard University  Bernard Goldstein, University of Pittsburgh |

**Participant Name Organization City, Country**

Roberto Accinelli Universidad Peruana Cayetano Heredia Lima, Peru

Heather Adair-Rohani World Health Organization (WHO) Geneva, Switzerland

Rajeev Ahuja Lok Nayak Hospital New Delhi, India

Dewan Alam ICDDR,B Dhaka, Bangladesh

Duane Alexander National Institutes of Health (NIH) Bethesda (MD), USA

Houmam Araj NIH Bethesda (MD), USA

Kwaku Poku Asante Kintampo Health Research Centre Kintampo, Ghana

Kalpana Balakrishnan Sri Ramachandra University Chennai, India

John Balbus NIH Bethesda (MD), USA

Kris Balderston Department of State Washington (DC), USA

John Balmes University of California Berkeley (CA), USA

Michael Bates University of California, Berkeley Berkeley (CA), USA

Jill Baumgartner University of Minnesota St. Paul (MN), USA

Jamie Bechtel New Course Bainbridge Island (WA), USA

Neil Bellefeuille The Paradigm Project Monument (CO), USA

Ron Bills Envirofit International Fort Collins (CO), USA

Linda Birnbaum NIH Durham (NC), USA

Simon Bishop Shell Foundation London, UK

Gerald Bloomfield Duke University Durham (NC), USA

Faustina Boakye World Vision Ghana Accra North, Ghana

Anita Botti Department of State Washington (DC), USA

Neal Brandes U.S. Agency for International Development Washington (DC), USA

Patrick Breysse Johns Hopkins University Baltimore (MD), USA

Robert Brook University of Michigan Ann Arbor (MI), USA

Nigel Bruce University of Liverpool Liverpool, UK

Sonia Buist Oregon Health & Science University Portland (OR), USA

Dana Charron Berkeley Air Monitoring Group Berkeley (CA), USA

William Checkley Johns Hopkins University Baltimore (MD), USA

Dong Feng Chen Harvard University Boston (MA), USA

Steven Chillrud Columbia University Palisades (NY), USA

Arun Chockalingam NIH Bethesda (MD), USA

Janine A. Clayton NIH Bethesda (MD), USA

Hillary Clinton (by video) Department of State Washington (DC), USA

Francis S. Collins NIH Bethesda (MD), USA

Gwen Collman NIH Research Triangle Park (NC), USA

Leslie Cordes Global Alliance for Clean Cookstoves Washington (DC), USA

Siân Curtis University of North Carolina at Chapel Hill Chapel Hill (NC), USA

David DeMarini Environmental Protection Agency (EPA) Research Triangle Park (NC), USA

Elisa Derby Winrock International Jamaica Plain (MA), USA

Jacqueline Devine World Bank Washington (DC), USA

Shane Diekman Centers for Disease Control and Prevention Atlanta (GA), USA

Gregory Diette Johns Hopkins University Baltimore (MD), USA

Kathie Dionisio Harvard University Jamaica Plain (MA), USA

Douglas Dockery Harvard University Boston (MA), USA

Rufus Edwards University of California, Irvine Irvine (CA), USA

Mahmoud El-Oteify Assiut University Hospital Assiut, Egypt

William Elwood NIH Bethesda (MD), USA

Henry Falk CDC (Consultant) Atlanta (GA), USA

Daniel Feikin Johns Hopkins University Baltimore (MD), USA

Matthew Fenton NIH Bethesda (MD), USA

Lawrence Fine NIH Bethesda (MD), USA

Paul Garbe CDC Atlanta (GA), USA

Josefina Garcia US Naval Medical Research Unit – 6 Peru Lima, Peru

Patricia Garcia Universidad Peruana Cayetano Heredia Lima, Peru

Armen Ghazarian NIH Bethesda (MD), USA

Amy Ginsburg PATH Seattle (WA), USA

Roger Glass NIH Bethesda (MD), USA

Bernard Goldstein University of Pittsburgh Pittsburgh (PA), USA

Jay Graham USAID Washington (DC), USA

Kimberly Gray NIH Research Triangle Park (NC), USA

Joseph Graziano Columbia University New York (NY), USA

Richard Grinnell HELPS International, Inc. Guatemala City, Guatemala

Alan Guttmacher NIH Bethesda (MD), USA

Nadia Hansel Johns Hopkins University Baltimore (MD), USA

Corinne Hart United Nations Foundation Washington (DC), USA

Patricia Hibberd Massachusetts General Hospital Boston (MA), USA

Virginia Hight University Research Co., LLC Bethesda (MD), USA

Philip Hopewell University of California, San Francisco San Francisco (CA), USA

Adnan Hyder Johns Hopkins University Baltimore (MD), USA

Darby Jack Columbia University New York (NY), USA

James Jetter EPA Research Triangle Park (NC), USA

James Kiley NIH Bethesda (MD), USA

Sylvester Ngetich Kimaiyo AMPATH Eldoret, Kenya

Patrick Kinney Columbia University Central Valley (NY), USA

David Lagat AMPATH Eldoret, Kenya

David Lee University of Miami Miami (FL), USA

Orin Levine Johns Hopkins University Baltimore (MD), USA

Edward Liechty Indiana University Indianapolis (IN), USA

Esther Lwanga USAID Washington (DC), USA

Yvonne Maddox NIH Bethesda (MD), USA

William J. Martin II NIH Bethesda (MD), USA

Margaret Matinga University of Johannesburg Johannesburg, South Africa

Gina McCarthy EPA Washington (DC), USA

Linda McCauley Emory University Atlanta (GA), USA

John McCracken Universidad del Valle de Guatemala San Lucas Sacatepequez, Guatemala

Sumi Mehta Global Alliance for Clean Cookstoves Washington (DC), USA

Richard Miller University of Rochester Rochester (NY), USA

John Mitchell EPA Washington (DC), USA

Charles Mock University of Washington Seattle (WA), USA

Kevin Mortimer Liverpool School of Tropical Medicine Liverpool, UK

Jacob Moss Department of State Washington (DC), USA

Srikanth Nadadur NIH Morrisville (NC), USA

Luke Naeher University of Georgia Athens (GA), USA

Clayton Nall Harvard University Cambridge (MA), USA

Yvonne Njage NIH Bethesda (MD), USA

Pilar Nores Bodereau Instituto Trabajo y Familia Lima, Peru

Saija Nurminen Embassy of Finland Washington (DC), USA / Finland

Seth Owusu-Agyei Kintampo Health Research Centre Kintampo, Ghana

Judy Palfrey Harvard University; Children’s Hospital Boston (MA), USA

Archana Patel Lata Medical Research Foundation Nagpur, India

Erin Patrick Women’s Refugee Commission New York (NY), USA

Piotr Pawlak Instituto Promundo Washington (DC), USA

Jennifer Peel Colorado State University Fort Collins (CO), USA

David Pennise Berkeley Air Monitoring Group Berkeley (CA), USA

Rogelio Perez-Padilla Instituto Nacional de Enfermedades Respiratorias Mexico City, Mexico

Bobbie Person CDC Decatur (GA), USA

Tamara Pilishvili CDC Atlanta (GA), USA

Daniel Pope University of Liverpool Liverpool, UK

Tony Punturieri NIH Bethesda (MD), USA

Sanjay Rajagopalan The Ohio State University Columbus (OH), USA

Tonse Raju NIH Bethesda (MD), USA

Stephen Rappaport University of California, Berkeley Berkeley (CA), USA

Britt Reid NIH Bethesda (MD), USA

Charles Rodes RTI International Research Triangle Park (NC), USA

Alyson Rose-Wood Department of Health and Human Services Washington (DC), USA

Christa Roth Food and Fuel Eschborn, Germany

Michael Sage CDC Atlanta (GA), USA

Amir Sapkota University of Maryland College Park (MD), USA

Debra Schaumberg Brigham & Women’s Hospital; Harvard University

Boston (MA), USA

Julie Schwaninger NIH Bethesda (MD), USA

Craig Shapiro Department of Health and Human Services Washington (DC), USA

Veena Sharma Self Employed Women’s Association Ahmedabad, India

Daniel Singer NIH Rockville (MD), USA

Kirk Smith University of California, Berkeley Berkeley (CA), USA

Justin Sosne Department of State Washington (DC), USA

Sarah Sowell EPA Washington (DC), USA

Robert Spengler CDC Atlanta (GA), USA

Dean Still Aprovecho Research Center Cottage Grove (OR), USA

Firehiwot Tachea San Jose State University San Jose (CA), USA

James Tielsch Johns Hopkins University Baltimore (MD), USA

Eser Tolunay NIH Bethesda (MD), USA

Alan Vette EPA Research Triangle Park (NC), USA

Martin Weber WHO Jakarta, Indonesia

Sheila West Johns Hopkins University Baltimore (MD), USA

Deborah Winn NIH Bethesda (MD), USA

Darrell Winner EPA Washington (DC), USA

Linda Wright NIH Rockville (MD), USA

Blair Wylie Harvard University; Massachusetts General Hospital Boston (MA), USA

Yawei Zhang Yale University New Haven (CT), USA
